# Tissue-specific changes in apoplastic proteins and cell wall structure during cold acclimation of winter wheat crowns

**DOI:** 10.1093/jxb/erx450

**Published:** 2018-01-24

**Authors:** Ian R Willick, Daisuke Takahashi, D Brian Fowler, Matsuo Uemura, Karen K Tanino

**Affiliations:** 1 Department of Plant Sciences, University of Saskatchewan, Saskatoon, SK, Canada; 2 United Graduate School of Agricultural Sciences, Iwate University, Morioka, Japan; 3 Department of Plant-biosciences and Cryobiofrontier Research Center, Faculty of Agriculture, Iwate University, Morioka, Japan

**Keywords:** Apoplast, cold acclimation, crown, FTIR, shotgun proteomics, winter wheat

## Abstract

The wheat (*Triticum aestivum* L.) crown is the critical organ of low temperature stress survival over winter. In cold-acclimated crowns, ice formation in the apoplast causes severe tissue disruption as it grows at the expense of intracellular water. While previous crown studies have shown the vascular transition zone (VTZ) to have a higher freezing sensitivity than the shoot apical meristem (SAM), the mechanism behind the differential freezing response is not fully understood. Cooling cold-acclimated crowns to –10 °C resulted in an absence of VTZ tetrazolium chloride staining, whereas the temperatures at which 50% of the SAM stained positive and 50% of plants recovered (LT_50_) were similar after cold acclimation for 21 (–16 °C) and 42 d (–20 °C) at 4 °C. Proteomic analysis of the apoplastic fluids identified dehydrins, vernalization-responsive proteins, and cold shock proteins preferentially accumulated in the SAM. In contrast, modifications to the VTZ centered on increases in pathogenesis-related proteins, anti-freeze proteins, and sugar hydrolyzing enzymes. Fourier transform infrared spectroscopy focal plane array analysis identified the biochemical modification of the cell wall to enhance methyl-esterified cross-linking of glucuronoarabinoxylans in the VTZ. These findings indicate that the SAM and VTZ express two distinct tissue-specific apoplastic responses during cold acclimation.

## Teaser

Changes in the apoplast associated with cell wall modifications and anti-freeze proteins in the shoot apical meristem and vascular transition zone suggest differential acclimation responses within the winter wheat crown.

## Introduction

A lack of adequate winter hardiness is the main environmental factor limiting winter wheat (*Triticum aestivum* L.) expansion into more northern latitudes (Fowler, 2012). Overwintering success is contingent on the degree of tolerance and/or avoidance within the crown to freezing—a combination of cold, mechanical, drought, and osmotic stresses (Levitt, 1980). Within the crown, mechanical damage from ice crystallization occurs first in the vascular transition zone (VTZ) located between the shoot apical meristem (SAM) and the nodal plate (Olien and Marchetti, 1976; Tanino and McKersie, 1985; Livingston *et al.*, 2013). Severe VTZ freezing injury impedes the regeneration of new roots, resulting in plant starvation and a delayed death response (Olien and Marchetti, 1976; Chen *et al.*, 1983). However, the interplay between the initial damage to the VTZ and a perceived lack of damage to the SAM is not fully understood.

Winter cereals pre-exposed to cold temperatures undergo a series of biochemical, biophysical, and molecular modifications, known as cold acclimation (Olien, 1965; Levitt, 1980; Griffith *et al.*, 1985, 2005; Livingston and Henson, 1998; Livingston *et al.*, 2006; Gusta *et al.*, 2009; Takahashi *et al.*, 2013*a*). In cold-acclimated plants, ice propagates in the apoplast and must remain in this space for the plant to avoid lethal intracellular freezing (Levitt, 1980). Despite evidence supporting different physiological (Chen *et al.*, 1983; Tanino and McKersie, 1985; Livingston *et al.*, 2013) and biochemical (Livingston and Henson, 1998; Livingston *et al.*, 2006) tissue-specific responses to freezing, most winter cereal cold acclimation studies treat the crown as a single unit (see review by Gusta *et al.*, 2009). Identification of differences in the cold-acclimated SAM and VTZ apoplastic space could explain differences in tissue-specific freezing injury, but has largely been unexplored.

The apoplast encompasses the primary cell wall, middle lamella, and intercellular air spaces. Type I cell walls contain equal amounts of cellulose and xyloglucans embedded in a pectin matrix (Carpita and Gibeaut, 1993; Hatfield *et al.*, 2016). Winter wheat and other members of *Poales* have type II cell walls composed of cellulose interlocked with glucuronoarabinoxylans, mixed linked β-glucans, and small quantities of pectin and xyloglucans (Carpita and Gibeaut, 1993; Carpita *et al.*, 2001; Hatfield *et al.*, 2016). As freezing tolerance is acquired, in both abscisic acid (ABA)-acclimated cell suspension cultures (Reaney *et al.*, 1989; Tanino et al., 1990, 1991) and cold-acclimated plants (Chen *et al.*, 1977; Griffith *et al.*, 1985; Weiser *et al.*, 1990; Rajashekar and Burke, 1996; Rajashekar and Lafta, 1996; Kubacka-Zębalska and Kacperska, 1999; Stefanowska *et al.*, 1999; Solecka *et al.*, 2008), cell walls increase in thickness and rigidity. Cell walls can be modified during the natural turnover of components (Carpita and Gibeaut, 1993; Hatfield *et al.*, 2016) or reconfigured by cell wall-modifying proteins (CWMPs) during cold acclimation (Weiser *et al.*, 1990; Takahashi et al., 2013a, b). In cold-acclimated winter oilseed rape (*Brassica napus* subsp. *oleifera* L.), pectin methylesterase cleaves methyl ester groups to promote calcium cross-linking and subsequently increases cell wall strength and rigidity (Kubacka-Zębalska and Kacperska, 1999; Stefanowska *et al.*, 1999; Solecka *et al.*, 2008). In type II cell walls, peroxidases oxidize phenolic compounds to synthesize radicals needed to dimerize cell wall covalent esterified linkages (Fry, 1986; Hatfield *et al.*, 2016). Glucuronoarabinoxylans are cross-linked with lignin to enhance wall rigidity by phenylpropanoid hydroxycinnamates such as ferulic acid (Fry, 1986; Carpita and Gibeaut, 1993; Domon *et al.*, 2013; Baldwin *et al.*, 2014; Hatfield *et al.*, 2016). In cold-acclimated cereals, the apoplastic space contains a range of solutes including arabinoxylans (Olien, 1965), fructans, and sucrose (Livingston and Henson, 1998) that modify ice crystal growth (Olien and Smith, 1977; Gusta *et al.*, 2004). Proteomic studies on apoplastic fluids collected from cold-acclimated winter cereals have also identified transport, oxidative response, pathogenesis-related, and anti-freeze proteins (AFPs) (Herman *et al.*, 2006; Takahashi *et al.*, 2013a, b) associated with enhanced freezing tolerance and inhibition of ice re-crystallization (Knight *et al.*, 1995; Griffith and Yaish, 2004).

The use of GC-MS has provided valuable information on cell wall composition (Fry, 1986; Carpita and Gibeaut, 1993; Carpita *et al.*, 2001; Domon *et al.*, 2013; Hatfield *et al.*, 2016). However, bulk sampling can unintentionally mask modifications to the cell wall. Fourier transform infrared spectroscopy (FTIR) coupled with a spectro-microscope addresses this problem by selecting a point of interest within the cell wall. Individual bonds associated with specific chemical functional groups have unique vibrational frequencies (see reviews by McCann *et al.*, 1997; Vijayan *et al.*, 2015). The infrared spectra-specific peak width and absorbance intensity can then be used to explain the relative difference in cell wall composition when exposed to environmental stress. In spring wheat kernels, FTIR coupled with a focal plane array (FPA) detector was used to identify frost injury to the cell wall (Xin *et al.*, 2013) and rachis node wall composition among fusarium head blight- (*Fusarium graminearum*) inoculated susceptible and tolerant cultivars (Lahlali *et al.*, 2016). Tanino *et al.* (2013) identified differences in pectin methyl esterification and protein secondary structure differences within the apoplastic space of cold-acclimated onion (*Allium fistulosum* L.) epidermal cells. By overlaying optical images of crown tissues with chemical information from infrared spectra, relative changes in cell wall composition can be characterized following cold acclimation.

Due to the vast number of potential factors involved in freezing tolerance and/or avoidance, a systematic approach is needed to identify important tissue-specific proteomic and biochemical changes localized with the apoplast. In the present study, we aimed to: (i) identify differences in the cold-acclimated SAM and VTZ apoplast using shotgun proteomics and (ii) contrast these proteomic changes with FTIR-FPA tissue level mapping in the crowns of winter wheat ‘Norstar’ (Grant, 1980). ‘Norstar’ was chosen because of its predominant role in breeding programs studying winter hardiness in the 1970s and 1980s, and its continued use as a control check in winter wheat freezing tests (Fowler, 2012).

## Materials and methods

### Plant growth conditions

Imbibed ‘Norstar’ seeds were held at 4 °C for 48 h. Seeds were transferred onto 14 × 24 cm polyethylene trays and then incubated in the dark at 21 °C for 48 h. Once roots were 1–2 cm in length, plants were transferred to hydroponic tanks and grown in aerated half-strength modified Hoagland’s solution as described by Chen *et al.* (1983). Tanks were transferred into a constant 20 °C growth chamber, with a 16 h day and photosynthetic photon flux density (PPFD) of 400 μmol m^−2^ s^−1^ until seedlings reached the third to fourth leaf stage. For cold acclimation, plants were transferred to a constant 4 °C growth chamber with a 16 h photoperiod and PPFD of 350 μmol m^−2^ s^−1^. Tanks were arranged in a completely randomized design, re-supplied with half-strength Hoagland’s solution every week, and re-filled with reverse osmosis water as required.

### Determination of freezing survival

Crowns acclimated for 0, 21, or 42 d were covered in moist sand in aluminum weighing cans and placed in a programmable freezer held at –4 °C for 12 h, and then cooled at 2 °C h^−1^ down to –28 °C as described by Chen *et al.* (1983) with the following modifications. Fifteen crowns were removed from the freezer at five pre-determined test temperatures at 2 °C intervals over a temperature range expected to bracket the LT_50_ (lethal temperature at which 50% of the plants recover). Crowns were thawed overnight at 4 °C and transplanted into pots containing Sunshine Mix #3 (Terralink Horticulture Inc., Abbotsford, BC, Canada) in a growth chamber maintained at 20 °C with a 16 h photoperiod and PPFD of 350 μmol m^−2^ s^−1^. Plants were rated for survival after 21 d. The LT_50_ was predicted from a survival versus temperature sigmoid curve. Determinations were repeated four times for each temperature treatment.

The TTC_50_, or the temperature at which 50% of the VTZ or SAM positively stained red, was determined for each acclimation interval (0, 21, and 42 d). Crowns were frozen as outlined above, but plants were removed at three sub-zero temperatures (at 2 °C intervals) around the estimated LT_50_ value. Twenty crowns were removed from the freezer (five per replicate) for each treatment temperature. After 7 d of recovery, crowns were washed in reverse osmosis water and then sectioned longitudinally. Sectioned crowns were stained with tetrazolium chloride (TTC) and viewed under a dissecting microscope as described by Tanino and McKersie (1985) and Livingston *et al.* (2013). Tissues were rated as viable if the entire SAM or VTZ stained positive for TTC. The experiment was repeated four times. Twenty fully acclimated crowns were removed following exposure to –12 °C for imaging during the first 7 d of recovery.

### Isolation of apoplastic fluids

The SAM and VTZ apoplastic fluids were extracted using a modified protocol as described by Boudart *et al.* (2005). For each of four replicates, excised SAM and VTZ tissue was collected from 20 plants and vacuum infiltrated in a syringe with 0.3 M sorbitol solution for 5 min. Crown tissues were blotted with an absorbent tissue, transferred into a 15 ml syringe packed with glass wool, and placed in a conical centrifuge tube. The syringe-in-tube apparatus was centrifuged in a swinging bucket at 6000 *g* at 4 °C for 2 h and the apoplastic fluids were collected as described by Gusta *et al.* (2004). The process was repeated three times and, for the final infiltration step, sorbitol was substituted with 0.2 M calcium chloride. Apoplastic fluid osmolarity was determined using a psychrometer (PsyPro, Wescor Inc., UT, USA) according to the manufacturer’s instructions.

For total protein extraction, 0.5 g of tissue was ground in a chilled mortar with extraction buffer [100 mM HEPES-KOH pH 8.0, 10 mM EDTA, 2 mM EGTA, 0.2% (v/v) Triton X-100, 1% (w/v) polyvinylpolypyrrolidone (PVPP), 2 mM DTT] in a 1:2 ratio. Samples were transferred to sterile microfuge tubes, centrifuged (15 min, 6000 *g* at 4 °C), and the supernatant was collected as described by Gregory *et al.* (2009). Total and apoplast extracts were filtered using Amicon Ultra 0.5 filters (molecular weight cut-off=3000; Millipore, Bedford, MA, USA) and assayed for protein concentration using the Bio-Rad (Berkley, CA, USA) protein assay kit according to the manufacturer’s instructions. To evaluate the degree of symplastic contamination in the apoplastic fluids, malate dehydrogenase (EC 1.1.1.37) activity was determined as described by Boudart *et al.* (2005). Duplicates were run, and four replicates were included for each experimental group.

### Sample preparation and data acquisition for nano-LC-MS/MS analysis

Prior to proteomic analysis, apoplastic fluids were fractionated using SDS–PAGE and visualized by silver staining (Kawamura and Uemura, 2003) to test for sample integrity. Samples were subjected to in-gel tryptic digestion for nano-LC-MS/MS analysis as described by Takahashi *et al.* (2013*a*). Peptide solutions were subjected to nano-LC-MS/MS analysis as described by Takahashi *et al.* (2013*a*) with the following modifications; spray voltage of 2.0 kV and collision-induced fragmentation was applied to the 10 most intense ions for identification of proteins at a threshold >500. The MS proteomics data have been deposited in the ProteomeXchange Consortium via the PRIDE partner repository (Vizcaíno *et al.*, 2016) with the data set identifier PXD007796 and 10.6019/PXD007796.

### Fourier transform infrared microscopy

Crowns were freeze-fixed in water and longitudinally sectioned using a cryo-microtome (Leica CM3050 S, Concord, Ontario, Canada). Sections (12 μm) were mounted on BaF_2_ polished circular discs (25 mm diameter, 1 mm thick; Crystran Ltd. Poole, UK) and placed in a desiccator for 48 h. Sections were imaged using a modified protocol as described by Tanino *et al.* (2013). Samples were maintained under a dry nitrogen purge to remove CO_2_ and H_2_O interference. Spectral maps were acquired using the following parameters: transmission mode, 4 cm^−1^ resolutions with 128 co-additions, 64 × 64 pixel mid-infrared FPA detector (Bruker Optics Inc., Milton, ON, Canada) connected to a Hyperion 3000 microscope using a 15× objective. Spectral maps were collected using Bruker Optics Opus software over the mid-infrared range (4000–900 cm^−1^). The most representative of the three separate maps were included in this study.

Principal component analysis on the fingerprint region (1800–900 cm^−1^) was performed using Minitab (version 16, State College, PA, USA). Score plots for the first two principal components were examined for spectra clustering. The percentage of variability in the data set was calculated for the two largest principal components. Protein secondary structure analysis of averaged spectra was carried out on 15 spectra from three separate biological samples in the amide I protein region (1700–1600 cm^−1^) using OPUS 7.2 as described by Lahlali *et al.* (2014).

### Statistical analysis

Analyses of freezing injury tests, osmoles, and protein concentrations were performed using the SigmaPlot 12.5 (Systat Software Inc., Chicago, IL, USA) two-way ANOVA procedure with Fisher’s least significant difference (LSD) test. Where applicable, the effects of acclimation time (0, 21, and 42 d) and tissue (SAM and VTZ) were considered as fixed effects, while repeated experiments, blocks, and replications within each block were considered as random effects.

For semi-quantitative analysis of apoplastic proteins, experimental raw MS/MS data were analyzed with Progenesis LC-MS software (version 4.0, Nonlinear Dynamics, Newcastle, UK). From the generated list of peptides, a reference run was selected and, based on this run, retention times for each subsequent run were aligned. Each feature was normalized based on the quantitative abundance ratio. Peptide abundances were compared using ANOVA (*P*<0.05), and a 2-fold threshold was used to determine increasing and decreasing proteins. Protein identification was conducted using the full peptide list with the MASCOT search engine (Matrix Science, London, UK) and the NCBInr Green Plants database utilizing the procedure outlined by Takahashi *et al.* (2013*a*). Protein information was exported from the Mascot xml format to Progenesis software which then associated protein information with the peptide results. Only proteins identified in all four biological replicates in each treatment group were considered in this analysis. Proteins identified were cross-referenced against NCBI protein blast (https://blast.ncbi.nlm.nih.gov/Blast.cgi?PAGE=Proteins) [accessed 4 January 2017], UniProtKB (http://www.uniprot.org/) [accessed 4 January 2017], TargetP 1.1 (http://www.cbs.dtu.dk/services/TargetP/index.php?infile=../prjanalysis/tmp/lotus49212.fsa) [accessed 23 December 2016], SOSUI/G (http://harrier.nagahama-i-bio.ac.jp/sosui/sosuiG/sosuigsubmit.html) [accessed December 23 2016], and Pred-GPI (http://gpcr.biocomp.unibo.it/predgpi/pred.htm) [accessed 4 December 2016] to determine protein function, subcellular localization, secretome association, as well as predictions on whether the protein is soluble or membrane bound and putative identification of glycophoaphatidylinositol (GPI)-anchored proteins. The annotated protein list is available in Supplementary Table S1 at the Dryad Digital Repository (http://dx.doi.org/10.5061/dryad.p65dp).

To estimate carbohydrate and methyl esterification contents, spectra were normalized using the protein peak (1700–1650 cm^−1^), whereas amide I and II peaks were normalized using the esterification peak (1740 cm^−1^). Estimation of individual components was determined by integrating the area under specific bands. Integrated peak areas were determined using the OPUS integration method C (Lahlali *et al.*, 2016). Significance in integrated peak areas was determined from 15 spectra collected from three biological samples. In instances where ANOVA detected significant differences, Fisher’s LSD test was used to determine significant differences among cold acclimation treatments (*P*<0.05).

## Results

### Tissue-specific differences in freezing survival resulting from length of cold acclimation treatment

**Fig. 1.**
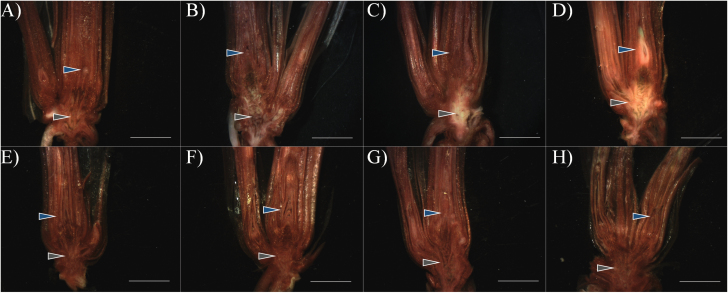
Tetrazolium chloride vital staining of hand-sectioned ‘Norstar’ winter wheat crowns observed after freezing to –12 °C. Plants that were cold acclimated for 42 d were placed in aluminum weighing cans filled with moist sand, held at –4 °C for ice nucleation, then cooled to –12 °C at a rate of –2 °C h^−1^ (A–D). A subsection of plants were not exposed to freezing temperatures to act as unfrozen controls (E–H). Frozen plants were thawed overnight and then both freeze-exposed and unfrozen controls were planted in potting mix and allowed to recover at 20 °C. Crowns were sampled after 1 (A and E), 3 (B and F), or 7 d (C, D, G, and H) of recovery and stained with tetrazolium. A red color identifies live cells/tissue that can reduce tetrazolium, while the off-white and yellow tissue is injured or dead as a result of freezing. After 7 d post-freezing, two patterns emerge, with either a loss of red coloration in the vascular transition zone (VTZ; C) or a loss of coloration within the VTZ and the shoot apical meristem (SAM; D). Blue arrows indicate the SAM, and gray arrows indicate the VTZ. White scale bar=0.6 cm.

‘Norstar’ crowns acclimated for 0, 21, and 42 d were subjected to a controlled freeze test and evaluated by regrowth and TTC staining (Fig. 1; Table 1). One day after freezing, an absence of TTC staining (off-white) was visualized near the nodal plate within the VTZ (Fig. 1A). Following 3 d of recovery, a clear demarcation between live and damaged tissues was visible (Fig. 1B), and the damaged region expanded to incorporate a greater proportion of the VTZ. By 7 d, recovering plants maintained the degree of damage to only the VTZ (Fig. 1C). Plants unable to recover lost their capacity to reduce TTC in the SAM and surrounding leaf sheath (Fig. 1D). Damage to the SAM and VTZ, as revealed by TTC staining, varied depending on the duration of cold acclimation. After 21 d or 42 d of cold acclimation, ‘Norstar’ TTC_50_ for VTZ was around –10 °C (Table 1). At 21 d or 42 d, the SAM TTC_50_ was significantly lower than the VTZ TTC_50_ (*P*<0.05). Furthermore, SAM TTC_50_ values at 21 d or 42 d were more comparable with whole-plant recovery LT_50_ values at 21 d or 42 d than VTZ TTC_50_ (Table 1).

**Table 1.** Whole-plant recovery and tetrazolium staining of the shoot apical meristem (SAM) and vascular transition zone (SAM) of ‘Norstar’ winter wheat after freezing

Acclimation (d)	LT_50_	TTC_50_	
		SAM	VTZ
0	–4.8 c	–3.6 c	–3.5 c
21	–15.6 b	–15.7 b	–9.5 b
42	–19.8 a	–19.9 a	–10.9 a
LSD_0.05_	2.1	1.6	0.5

Determination of the temperature at which 50% of the population was unable to recover from freezing (LT_50_) and the temperature at which 50% of the ‘Norstar’ crown SAM or VTZ tissues stained positive for tetrazolium chloride vital staining (TTC_50_). Plants were acclimated for 0, 21, or 42 d at 4 °C.

Means followed by the same letter within each column are not significantly different based on Fisher’s LSD test (*P*<0.05) and were calculated from four independent tests.

The tissue-specific osmotic potential of the apoplastic fluids varied significantly based upon tissue and acclimation time (*P*<0.05; Table 2). Irrespective of the duration of cold acclimation, osmotic potential decreased significantly in both the SAM and VTZ as compared with fluids collected at 0 d of acclimation (*P*<0.05). While there was no significant difference in osmotic potential between fluids collected in the SAM and VTZ after 21 d (*P*>0.05), there was a significantly greater osmotic potential in apoplastic fluids collected from the SAM compared with the VTZ after 42 d of cold acclimation (*P*<0.05).

**Table 2.** The osmotic potential of apoplastic fluids collected from ‘Norstar’ shoot apical meristem (SAM) and vascular transition zone (VTZ) following 0, 21, or 42 d of cold acclimation

Tissue	Time (d)	Osmotic potential (MPa)
SAM	0	–1.91 c
	21	–2.13 b
	42	–2.19 a
VTZ	0	–1.97 c
	21	–2.11 b
	42	–2.11 b
LSD_0.05_		0.05

Means followed by the same letter within each column are not significantly different based on Fisher’s LSD test (*P*<0.05).

### Subcellular localization and functional classification of proteins

**Fig. 2.**
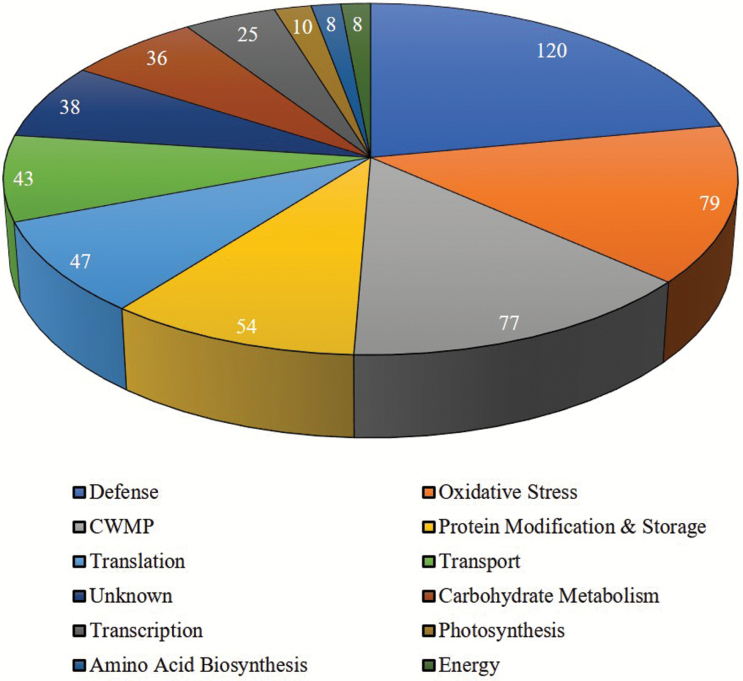
Functional categorization of all apoplast proteins identified in the SAM and VTZ apoplast following exposure to 0, 21, and 42 d of cold acclimation. Apoplastic proteins were classified into 12 functional categories based on a modified definition proposed by Boudart *et al.* (2005).

Malate dehydrogenase activity, an indicator of symplastic contamination, was <0.5% in all apoplast samples (Supplementary Table S2 at Dryad). To investigate proteome alterations during cold acclimation (21 d or 42 d) in the SAM and VTZ, changes in the presence and abundance of apoplastic proteins were quantified using the shotgun proteomic technique. A total of 545 proteins were identified in all tissues and treatments. Of the proteins with a putative subcellular localization (422 in total), 72% (305 proteins) were identified to be extracellular or plasma membrane proteins and 29 were identified as putative GPI-anchored proteins. Of the classified proteins (343 in total), 75% were determined to have the putative secretory capacity (Supplementary Table S1 at Dryad). Proteins were classified into 12 functional categories (Fig. 2). The majority were classified as defense (120 proteins, 22.0%), oxidative stress response (79 proteins, 14.5%), CWMPs (77 proteins, 14.1%), and transport (43 proteins, 7.9%) proteins.

### Relationship between SAM and VTZ cold-acclimated proteins

**Fig. 3.**
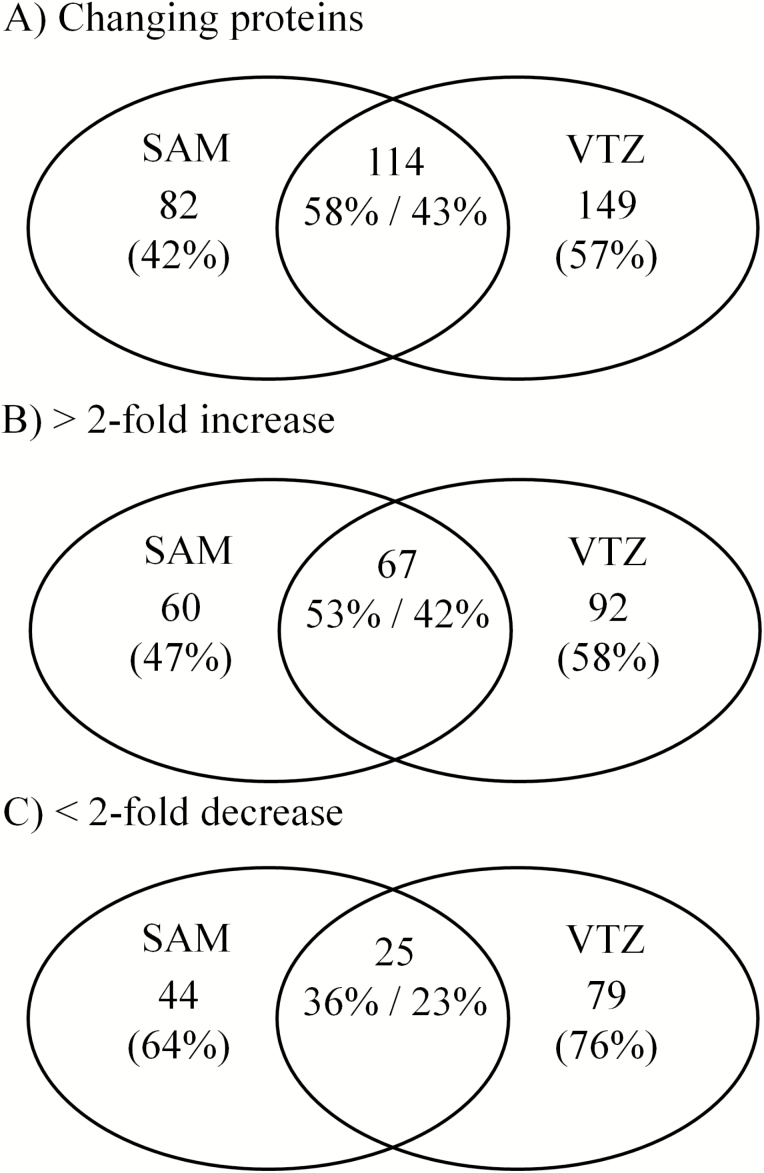
Venn diagrams of changed (A), increasing (B), or decreasing (C) apoplast proteins when contrasting abundance in the SAM and VTZ at 42 d and 21 d of cold acclimation. Venn diagrams were generated using http://bioinfogp.cnb.csic.es/tools/venny/.

When we considered apoplast proteins showing responses in the SAM or VTZ irrespective of the duration (21 d and 42 d) and direction (increased or decreased), ~20% of proteins only responded in the SAM while 11% were identified to change in response to cold only in the VTZ (Supplementary Fig. S1 at Dryad). The proportions of proteins in the SAM or VTZ increased along with the duration of cold acclimation (Supplementary Fig. S1 at Dryad; Fig. 3). The percentage of tissue-specific apoplast proteins from 42 d and 21 d fractions induced in the SAM (47%) and VTZ (58%) was higher than the 0 d acclimation in the SAM (23% or 35%) and VTZ (28% or 38%; Fig. 3B; Supplementary Fig. S1 at Dryad). Similar trends were observed with proteins of decreasing abundance (Fig. 3C; Supplementary Fig. S1 at Dryad).

### Functional categorization of SAM and/or VTZ cold-responsive proteins

In general, the proportions of cold-responsive proteins within each functional category at 21 d or 42 d compared with 0 d of acclimation at 4 °C were similar in the SAM and VTZ (Supplementary Fig. S1 at Dryad). This was consistent with the results of whole apoplast proteins illustrated in Fig. 2. As was the case with the whole apoplast proteome, defense, CWMP, oxidative stress, protein modification, and storage were the predominant categories in SAM and VTZ (Supplementary Fig. S1 at Dryad).

Differences in SAM and VTZ proteins from the four functional categories (defense, oxidative response, CWMP, and transport) were identified when contrasting 42 d and 21 d acclimation apoplastic fluid fractions. Of CWMP proteins, a greater proportion increased instead of decreased in the SAM (23 increased, 8 decreased) compared with the VTZ (19 increased, 17 decreased). The ratio of increased to decreased proteins was greater in the VTZ when focusing on defense (51 increased, 19 decreased) or transport (22 increased, 4 decreased) compared with SAM defense (33 increased, 22 decreased) or transport (11 increased, 2 decreased) apoplast proteins (Supplementary Table S1 at Dryad). When comparing 42 d and 21 d apoplast fluid fractions, there was no difference in the ratio of increased to decreased oxidative response proteins in the SAM (32 increased, 6 decreased) and the VTZ (36 increased, 7 decreased). Studying these proteins only as functional groups can mask certain trends within the cold-acclimated SAM and VTZ apoplast. Among CWMPs, invertases and fructan exohydrolases preferentially accumulated overall within the VTZ. Cold-responsive proteins of interest associated with transport and CWMP (Table 3) as well as defense and oxidative stress response proteins (Table 4) were identified in the SAM and VTZ.

**Table 3.** Selected cell wall-modifying proteins (C) and transport (T) apoplast proteins that are significantly increasing or decreasing in response to cold in the shoot apical meristem (SAM) or vascular transition zone (VTZ)

Accession	Description	Mean normalized abundance^a^			
		SAM 21 d	SAM 42 d	VTZ 21 d	VTZ 42 d
BAG72143.1	Alpha-glucosidase^C^	14734 (2.6)	13227 (2.3)	6616 (4.1)	7773 (4.8)
EMS58120.1	Alpha-xylosidase^C^	15 037 (1.9)	15 702 (2.0)	6755 (2.3)	10 518 (3.6)
CAD58960.1	Apoplastic invertase 1^C^	9670 (48.2)	7328 (36.4)	2707 (99.9)	734 (27.1)
AAM13694.1	Beta-d-glucan exohydrolase^C^	55 931 (3.4)	86 623 (5.3)	20 123 (4.3)	16 124 (3.5)
AJA71651.1	Beta-expansin 1a^C^	31 (nd)	95 (nd)	293 (nd)	320 (nd)
XP_003579704.1	Beta-fructofuranosidase 2-like^C^	12084 (134.8)	9069 (101.2)	1654 (86.5)	404 (21.1)
AAS97960.1	Cell wall beta-glucosidase^C^	16 611 (2.7)	29 388 (4.7)	5655 (8.1)	5143 (7.3)
BAM74038.1	Cell wall invertase^C^	159 (0.1)	1099 (0.7)	1832 (34.5)	4554 (85.6)
BAM74037.1	Cell wall invertase^C^	9240 (62.9)	7658 (52.1)	2485 (78.4)	602 (19.0)
AAS48872.1	Expansin EXPA3^C^	1848 (0.7)	4211 (1.7)	4922 (2.6)	11 042 (5.9)
ABI95405.1	Fasciclin-like protein FLA15^C^	19 037 (2.0)	29 350 (3.1)	8173 (3.7)	8054 (3.6)
BAE44509.1	Fructan exohydrolase^C^	3327 (6.9)	5865 (12.2)	2161 (5.9)	4170 (11.5)
EMT31770.1	Glucan endo-1,3-beta-glucosidase 13^C^	141 (1.1)	612 (4.8)	613 (0.9)	138 (0.2)
ABB90546.1	Lipid transfer protein^C^	1896 (nd)	2727 (nd)	608 (30.2)	4022 (200.1)
EAY95514.1	Lipid transfer protein OsI_17360^C^	16 511 (4.0)	55 580 (13.6)	10 708 (4.4)	12 388 (5.1)
AAG27707.1	Lipid transfer protein precursor^C^	30 867 (1.5)	59 259 (2.8)	36 639 (1.3)	21 9881 (7.9)
AAK20395.1	Lipid transfer protein precursor^T^	33 750 (1.3)	63 712 (2.5)	40 958 (0.9)	199 771 (4.4)
BAJ96826.1	Lipid transport predicted protein^T^	2615 (9.6)	10 157 (37.3)	6133 (3.6)	53 984 (31.9)
EMT05022.1	Pectinesterase 3^C^	3454 (1.6)	5378 (2.4)	2362 (7.1)	3691 (11.1)
BAJ98765.1	Pectinesterase inhibitor^C^	7570 (76.6)	8092 (81.9)	751 (0.5)	2508 (1.8)
EMT00190.1	Putative polygalacturonase^C^	17 648 (0.6)	34 941 (1.1)	24 208 (1.9)	85 193 (6.6)
CAH69206.1	Type 1 non-specific lipid transfer protein^T^	5200 (8.3)	14 520 (23.1)	3281 (2.5)	12 196 (9.2)
AAC49404.1	WCOR719^C^	30 160 (63.0)	17 909 (37.4)	10 625 (9.3)	5237 (4.6)
ABU55395.1	Xylanase inhibitor 602OS^C^	217 779 (2.1)	328 160 (3.1)	215 744 3.0)	543 555 (7.7)

a Values in parentheses are the fold change difference from 0 d. Instances where proteins were not identified were indicated in parentheses as not detected (nd).

**Table 4.** Selected defense (D) and antioxidant response (R) apoplast proteins that are significantly increasing or decreasing in response to cold in the shoot apical meristem (SAM) or vascular transition zone (VTZ)

Accession	Description	Mean normalized abundance^a^			
		SAM 21 d	SAM 42 d	VTZ 21 d	VTZ 42 d
ACT65562.1	70 kDa heat shock protein^D^	5097 (21.3)	3514 (14.7)	21 (0.9)	69 (3.0)
AAD09343.1	ABA-responsive protein^D^	3327 (4.1)	5164 (6.4)	5404 (2.0)	14134 (5.3)
EMS52349.1	Cationic peroxidase SPC4^R^	16 333 (2.8)	28 670 (5.0)	25 309 (1.0)	132 178 (5.4)
XP_003580049.1	Chitinase 5-like^D^	0 (nd)	0 (nd)	1093 (0.6)	5969 (3.2)
BAD06324.1	Cold shock domain protein 2^D^	0 (nd)	1225 (nd)	0 (nd)	750 (24.6)
EMS48364.1	Cold shock protein CS66^D^	4747 (nd)	2332 (nd)	0 (nd)	0 (nd)
CAC12881.1	Cold-regulated protein^D^	1718 (17.2)	1731 (17.4)	5756 (87.3)	14340 (217.5)
XP_015631567.1	Cucumisin-like serine protease^D^	2900 (4.9)	8564 (14.4)	225 (0.3)	1646 (1.9)
BAB18766.1	Cysteine proteinase inhibitor^D^	98 673 (46.7)	51 758 (24.5)	64 001 (32.1)	77 628 (39.0)
BAJ89304.1	Defensin^D^	8221 (6.7)	7463 (6.1)	15 743 (34.9)	23 273 (51.5)
BAC10287.1	Defensin^D^	41 633 (9.4)	38 121 (8.6)	52 798 (52.2)	50 127 (49.6)
CAI65403.1	Dehydrin^D^	18 956 (80.0)	5764 (24.3)	27 338 (45.9)	2177 (3.7)
AAY16796.1	Early salt stress/cold acclimation- induced protein 2-3^D^	651 (0.8)	5826 (7.5)	3432 (1.3)	11 224 (4.2)
XP_014753148.1	GDSL esterase/lipase^D^	13 303 (2.2)	9482 (1.5)	10 075 (66.9)	5666 (37.6)
AAG00426.1	Germin B^D^	87 348 (0.9)	230 880 (2.3)	120 156 (2.0)	313 249 (5.2)
BAD10332.1	Glutaredoxin^R^	21 740 (58.4)	19 880 (53.4)	16 047 (65.5)	25 233 (103.0)
Q43472.1	Glycine rich RNA-binding protein^D^	195 887 (19.8)	102 286 (10.3)	92 000 (16.7)	62 158 (11.3)
EMT06661.1	Monothiol glutaredoxin-S10^R^	5768 (36.1)	4481 (28.0)	3196 (85.6)	6118 (163.8)
EMT19353.1	Peroxidase 70^R^	4520 (31.0)	13 652 (93.7)	19 792 (13.6)	112 959 (77.6)
EMS57180.1	Subtilisin-like protease^D^	1084 (184.3)	1146 (194.8)	2 (0.0)	20 (0.2)
EMT30759.1	Subtilisin-like protease^D^	637 (0.5)	1869 (1.6)	3204 (5.7)	9040 (16.2)
BAJ85823.1	Thaumatin^D^	80 492 (15.1)	65 217 (12.2)	52 439 (10.9)	17 965 (3.7)
EMT27591.1	Thaumatin^D^	210 (0.6)	459 (1.3)	3637 (2.2)	21 071 (12.6)
ACV20868.1	Thioredoxin-dependent peroxidase^R^	25110 (10.9)	11478 (5.0)	4904 (253.0)	8987 (463.6)
AAB18208.1	WCOR 615^D^	8289 (568.5)	7040 (482.8)	259 (nd)	14 (nd)
BAK07537.1	Wheat cold induced 16-like^D^	5922 (6280.0)	3405 (3610.5)	65 (2.6)	280 (11.3)

a Values in parentheses are the fold change difference from 0 d. Instances where proteins were not identified are indicated in parentheses as not detected (nd).

### Identification of bands of interest in the FTIR spectra

**Fig. 4.**
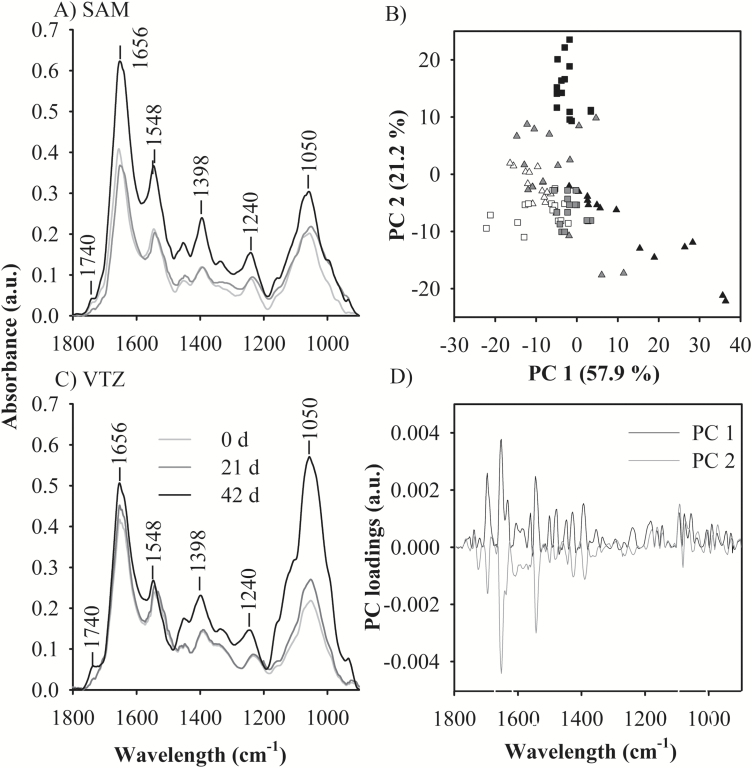
Application of principal components analysis to the fingerprint region (1800–900 cm^−1^) of Fourier-transformed infrared (FTIR) spectra collected from the apoplast to define differences in cold-acclimated ‘Norstar’ crown tissues. Plants were exposed to 0, 21, or 42 d of cold acclimation; crowns were then harvested and freeze sectioned for focal plane array-FTIR imaging. Averaged infrared spectra were collected from two regions of interest: (A) the shoot apical meristem (SAM) and (B) the vascular transition zone (VTZ). (C) Within the score plot, the relative abundance of the first two principal components (PCs) for 15 individual co-added spectra for each tissue and treatment group account for ~79 % of the total variation in each population. Spectra correspond to samples taken from the SAM (triangle) and VTZ (square) of 0 (white), 21 (gray), or 42 d (black) acclimated crowns. (D) An enrichment in the PC1 and PC2 loading values at ~1650 cm^−1^ indicates higher amounts of protein by characteristic peaks of wave numbers in the fingerprint region (reviewed by Vijayan *et al.*, 2015). The positive PC1 and negative PC2 value at 1650 cm^−1^ within the loading plot corresponds to spectra from 42 d SAM in the score plot. The highest positive PC2 peak corresponds to a loading value of ~1050 cm^−1^ which corresponds to the carbohydrates and spectra from the 42 d VTZ score plot.

Bands associated with different functional groups within the fingerprint region (1800–900 cm^−1^) such as carbonyl esterification (C=O stretching at 1740 cm^−1^), amide I (C=O stretching, and N-H bending of protein peptide bonds between 1700 cm^−1^ and 1600 cm^−1^) and glucuronoarabinoxylan (C-O-C stretching of glycosidic bonds at 1050 cm^−1^ and C-C ring vibration at 995 cm^−1^) were identified (Fig. 4A, C) and assigned as described previously (McCann *et al.*, 1997; Vijayan *et al.*, 2015). Spectra were deconstructed into individual data points, and variability in the fingerprint region was analyzed using principal component analysis. Up to 79% of the total variation amongst all spectra were explained by the first two principal components.

### Protein and secondary structures

**Fig. 5.**
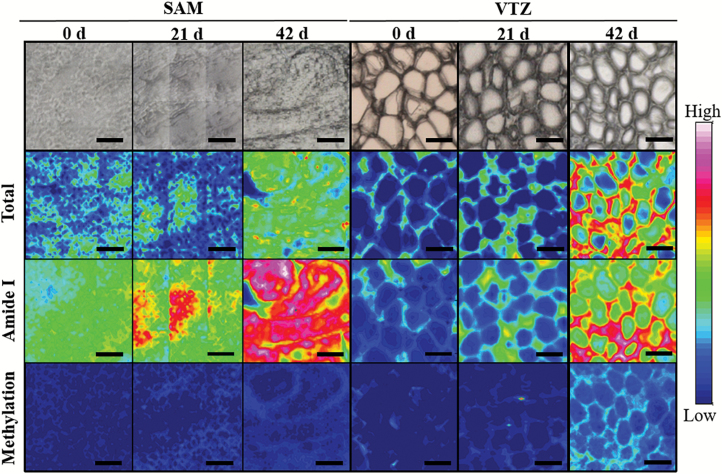
Comparisons between chemical maps of a ‘Norstar’ crown transverse section using Fourier transform infrared (FTIR) spectroscopy and imaging using a 64 × 64 focal plane array detector on an FTIR microscope. Sections were taken from plants exposed to 0, 21, and 42 d of cold acclimation. Images are representative of sections taken from one of three biological crown replicates. Each individual micrograph is comprised of nine separate images captured sequentially using a motorized stage and OPUS software. The first row of images shows a light micrograph of the mapped section. The second row of images shows the micrograph overlaid with a false-color map created with infrared spectroscopy using the integrated absorbance of the spectral region between 4000 cm^−1^ and 900 cm^−1^. The third row of images shows the micrograph overlaid with infrared spectroscopy using the integrated absorbance of the spectral region between 1700 cm^−1^ and 1580 cm^−1^ (amide I), attributed to protein. The third row of images shows the micrograph overlaid with infrared spectroscopy using the integrated absorbance of the spectral region between 1760 cm^−1^ and 1700 cm^−1^, attributed to the degree of methyl esterification. A rainbow scheme has been used to denote absorbance, with the warmest colors (red) indicating the highest absorbance, while cool colors (blue) represent a low spectral intensity. White scale=50 μm.

The positive influence of principal component one loadings and the negative influence of principal component two corresponded to peak values around amide I (1656 cm^−1^) when the score plot (Fig. 4B) was compared with the loading plot (Fig. 4D). In the score plot, 42 d SAM spectra coalesce as an individual group due to amide I peak intensity (Fig. 4B). Amide I integrated absorption peak areas were significantly different based upon temperature treatment (*P*<0.05; Table 5; Fig. 5). At 42 d of cold acclimation, amide I absorption peak areas were significantly higher (*P*<0.05) in the SAM compared with the VTZ.

**Table 5.** Integrated absorption spectra for methyl esterification (carbonyl ester, 1740 cm^−1^), protein (amide I, 1650 cm^−1^), and carbohydrates (glycosidic bond, 1050 cm^−1^) regions collected from cold-acclimated (0, 21, or 42 d at 4 °C) shoot apical meristem (SAM) and vascular transition zone (VTZ) crown tissues of ‘Norstar’ winter wheat

Tissue	Time (d)	Peak area (a.u.)		
		Methyl esterification	Protein	Carbohydrates
SAM	0	0.2 c	13.9 d	13.0 e
	21	0.4 c	24.1 c	17.7 b
	42	0.4 c	39.8 a	23.4 d
VTZ	0	0.3 c	14.8 d	22.1 c
	21	2.6 b	22.7 c	24.7 b
	42	4.1 a	35.6 b	49.1 a
LSD_0.05_		0.2	3.4	2.9

Areas beneath each peak were quantified using OPUS software. Each treatment group represents the mean value of 15 spectra.

Means followed by the same letter within each column are not significantly different based on Fisher’s LSD test (*P*<0.05).

Absorbance peaks observed at 1656 cm^−1^ and 1638 cm^−1^ (Fig 4) within the amide I region were previously identified in plant tissue as protein secondary structures (Lahlali *et al.*, 2014). Smaller individual peaks were visible in the second derivative of the absorbance spectra (Supplementary Fig. S2 at Dryad). Peaks associated with secondary structures were assigned to loops or β-turns (1662–1678 cm^−1^), α-helices (1648–1660 cm^−1^), β-sheets (1630–1640 cm^−1^), or random side chains (1610–1620 cm^−1^) as described by Lahlali *et al.* (2014). These band positions were used in curve fitting analysis as a resolution enhancement technique to separate overlapping bands. Differences in the α-helical and β-sheet peak area were noted due to acclimation treatment in the SAM and VTZ. The β-sheet peak area significantly increased (*P*<0.05) in the VTZ in comparison with the SAM after 42 d of cold acclimation. The α-helical peak area significantly increased (*P*<0.05), irrespective of tissue, due to cold acclimation (Table 6). The ratio of α-helical to β-sheet peak area significantly increased (*P*<0.05) in the 21 d and 42 d spectra, irrespective of tissue, compared with 0 d.

**Table 6.** Protein secondary structure absorbance peak areas (a.u.) identified from cold-acclimated (0, 21, or 42 d at 4 °C) shoot apical meristem (SAM) and vascular transition zone (VTZ) crown tissues of ‘Norstar’ winter wheat

Tissue	Time (d)	Peak area (a.u.)				
		α-Helix	β-Sheet	β-Turn	R-coil	α:β ratio^a^
SAM	0	0.14 c	0.14 d	0.15 c	0.08 c	1.00 b
	21	0.22 b	0.16 c	0.11 d	0.13 b	1.26 a
	42	0.29 a	0.19 b	0.11 d	0.16 a	1.63 a
VTZ	0	0.15 c	0.16 c	0.27 a	0.11 bc	1.00 b
	21	0.23 b	0.16 c	0.26 a	0.09 c	1.50 a
	42	0.31 a	0.23 a	0.19 b	0.14 ab	1.43 a
LSD_0.05_		0.03	0.02	0.02	0.03	0.25

Smaller peaks were identified within the amide I band when observing the spectra’s second derivative (Supplementary Fig. S2 at Dryad).

Curve-fitting analysis was conducted using OPUS software to identify and quantify each individual peak area.

Bands associated with each peak were assigned as described by Lahlali *et al.* (2014). Each treatment group represents the mean value of 15 spectra.

Means followed by the same letter within each column are not significantly different based on Fisher’s LSD test (*P*<0.05).

a α-helix to β-sheet ratio

### Carbohydrate and methyl esterification

Visible increases in FTIR spectral peaks associated with enhanced methyl esterification and glucuronoarabinoxylans were noted in the VTZ of cold-acclimated crowns but not the SAM. The integrated carbohydrate peak was the most significant at 42 d of acclimation in the VTZ (Fig. 4B, C;  Table 5). When contrasting the principal component score plots (Fig. 4B) with the loading plot (Fig. 4D), the most intense peak in principal component two had a positive influence on the score plot at ~1050 cm^−1^ and was associated to the greatest degree with spectra collected from 42 d VTZ. Integration at 1050 cm^−1^ indicates a significant increase in peak area as a result of cold acclimation, with significantly greater peaks associated with spectra collected from the VTZ compared with the SAM. Visible increases in the C=O vibration of the esterification peak at 1740 cm^−1^ and a peak of medium intensity at 1240 cm^−1^ were noted in VTZ spectra and in false-color FTIR-FPA chemical maps after 42 d of cold acclimation (Fig. 5). Integration of the SAM and VTZ spectra indicates significant increases in the esterification peak area as a result of 42 d or 21 d of cold acclimation in the VTZ compared with the SAM (Table 5).

## Discussion

### Differential freezing damage in the SAM and VTZ

In the present study, TTC vital staining showed that the TTC_50_ for the VTZ was near –10 °C in both 21 d and 42 d acclimated crowns, while the SAM TTC_50_ was comparable with the LT_50_ at –16 °C and –20 °C (Fig. 1; Table 1). This supports the theory that the VTZ in winter wheat is more susceptible to freezing damage at a slow cooling rate (2 °C h^−1^) (Chen *et al.*, 1983; Tanino and McKersie, 1985; Livingston *et al.*, 2013) and could act as a putative ice sink. In overwintering tree buds, scales act as ice sinks, allowing for the slow desiccation and survival of critical floret tissues (Ishikawa and Sakai, 1981). Damage to the VTZ was not lethal if the expansion of ice crystals did not completely sever the vasculature linking the SAM with the VTZ in winter oat (Olien and Marchetti, 1976) or if cells responsible for root regrowth associated with the VTZ remained intact in winter wheat (Chen *et al.*, 1983).

### Pathogenesis-related and AFP accumulation

Fifty-five putative AFPs or pathogen-related proteins were identified in this study, with 31 responding in the VTZ (26 increased, 5 decreased) while 23 changed in the SAM (17 increased, 6 decreased) when comparing protein abundance at 42 d and 21 d (Supplementary Table S1 at Dryad). Increases in β-sheet secondary structures, as noted in the cold-acclimated VTZ (Table 6), have been associated with AFP ice-binding sites purified from perennial ryegrass (*Lolium perenne* L.; Middleton *et al.*, 2009) and carrot (*Daucus carota* L.; Worrall *et al.*, 1998). Pathogenesis-related β-1,3-glucanases, endochitinases, and thaumatins synthesized during cold acclimation have cold acclimation capacity to control and inhibit ice crystal growth (Hon *et al.*, 1995; Chun *et al.*, 1998; Griffith and Yaish, 2004; Griffith *et al.*, 2005), degrade pathogen cell walls, and inhibit fungal enzymes (Hiilovaara-Teijo *et al.*, 1999; Griffith and Yaish, 2004).

Chun *et al.* (1998) concluded that apoplastic AFPs may not be influenced by the coldest lethal temperatures in plants, but through potentially lethal processes that occur at warmer sub-zero temperatures. While snow insulates the crown against low sub-zero temperatures, it also maintains ambient soil temperatures near the melting point of plant tissues where ice re-crystallizes to form large masses of ice (Knight *et al.*, 1995; Griffith and Yaish, 2004). In cold-acclimated crowns, formation of large ice crystals disrupts tissue structure in the VTZ (Olien and Smith, 1977; Tanino and McKersie, 1985; Livingston *et al.*, 2013). Since the VTZ is the largest source of free water in winter wheat crowns (Gusta, 1975), and is more sensitive to freezing than the SAM (Tanino and McKersie, 1985), a higher abundance of VTZ AFPs would be necessary to buffer against ice re-crystallization over winter.

Maintenance of a constant near freezing temperature and increased humidity under snow cover increases the propagation of psychrophilic snow molds (Hiilovaara-Teijo *et al.*, 1999; Griffith and Yaish, 2004). The VTZ is further stressed by pathogenic attack in cavities left by melted ice crystals (Olien and Smith, 1977; Livingston *et al.*, 2006). This can result in the browning phenomena within the crown associated with the formation of necrotic lesions and increased probability of plant death. Mechanical damage caused by ice propagation and the degeneration of VTZ cells during recovery also results in the formation of abscesses targeted by psychrophilic plant pathogens (Olien and Marchetti, 1976). Accumulation of antifungal proteins during cold acclimation within the VTZ (Table 4) could account for the acquisition of a systemic but non-specific defense against pathogenic stress (Hiilovaara-Teijo *et al.*, 1999; Griffith and Yaish, 2004).

### Vernalization-associated proteins

In the present study, a cold-regulated protein (CAC12881.1) was identified to have 93.9% homology with WCOR18 and increased in abundance to a greater degree in the SAM after 42 d of cold acclimation. WCOR18 was previously reported to be expressed under prolonged cold stress conditions, tied to floral development, and a marker for vernalization (Rinalducci *et al.*, 2011). A wheat cold shock protein 2 (WCSP2; BAD06324.1), associated with apical meristem floral development (Sasaki *et al.*, 2013), was present after 42 d of cold acclimation and in a higher abundance in the SAM compared with the VTZ (Supplementary Table S1 at Dryad). As was the case with the present study, Sasaki *et al.* (2013) observed that the level of WCSP2 was below detectable limits in non-acclimated plants, but gradually increased during cold acclimation and reduced freezing tolerance in Arabidopsis (*Arabidopsis thaliana*) mutants when overexpressed. How WCOR18 and WCSP2 are linked to the developmental control of vernalization is currently unknown.

### Membrane and lipid transfer proteins

Alterations to plasma membrane proteins during cold acclimation have been characterized using proteomic approaches (Kawamura and Uemura, 2003; Boudart *et al.*, 2005; Takahashi *et al.*, 2013*a, b*, 2016), and the maintenance of membrane integrity has been recognized as a critical mechanism to freezing survival (Steponkus, 1984; Uemura *et al.*, 2006). Previous reports using the calcium and sorbitol infiltration technique have identified extraction of apoplast and membrane-associated proteins (Boudart *et al.*, 2005; Takahashi *et al.*, 2016). Four late embryogenesis abundant family proteins, dehydrin (CAI65403.1), WCS66 (EMS48364.1), WCOR 615 (AAB18208.1), and wheat cold-induced 16 (BAK07537.1), were found to increase preferentially in the SAM in response to cold (Table 4). Proteins with dehydrin-like properties have been identified in association with the plasma membrane (Danyluk *et al.*, 1996). If the VTZ acts as an ice sink relative to the SAM, then increased abundance of SAM dehydrins could be one mechanism to reduce interaction between membrane bilayers during dehydration (Steponkus, 1984; Danyluk *et al.*, 1996; Uemura *et al.*, 2006) and maintain membrane stability throughout thawing (Chen *et al.*, 2013).

The majority of the 19 cold-increased lipid transfer proteins were found in the VTZ (Table 3; Supplementary Table S1 at Dryad). Lipid transfer proteins have numerous roles in response to cold acclimation, including increased soluble sugar content (Guo *et al.*, 2013), psychrophilic pathogen inhibition (Hon *et al.*, 1995; Griffith and Yaish, 2004), and higher thermal hysteresis and ice re-crystallization inhibition (Doxey *et al.*, 2006). Lipid transfer proteins are also involved in the deposition of suberin (Edstam *et al.*, 2013), which has been identified as a putative component of a post-freeze barrier surrounding the VTZ (Livingston *et al.*, 2013).

### Modification of sugar side chains by apoplastic invertases

Increased abundance of apoplastic and extracellular glucosidases, invertases, and fructan exohydrolases was observed to a greater degree in cold-acclimated VTZ compated with SAM apoplastic fluids (Table 3). Sugar-hydrolyzing enzymes are responsible for cleaving cell wall-bound arabinoxylans as well as sucrose and fructan side chains, increasing unbound extracellular sugar concentrations (Livingston and Henson, 1998; Zabotin *et al.*, 1998). High concentrations of sugar-hydrolyzing enzymes result in oligosaccharin accumulation during hemicellulose turnover and the acquisition of cold hardiness in winter wheat, and account for the presence of free arabinoxylans in the extracellular space as well as the postulated increase in cell ABA signaling (Zabotin *et al.*, 1998). Application of ABA significantly increased [U-^14^C]sucrose uptake into the cell, with the largest fraction partitioned to the insoluble fraction of the cell wall (Tanino *et al.*, 1990). Higher concentrations of soluble carbohydrates such as arabinoxylans or sucrose in the VTZ prevent ice adhesions and provide additional forms of protection to cell walls (Olien and Smith, 1977) and the plasma membrane (Uemura *et al.*, 2006).

### Modification to the cell wall matrix

In the present study, FTIR peaks associated with glucuronoarabinoxylans in VTZ tissues more than doubled after 42 d of cold acclimation and were 1.5 times greater in comparison with 42 d SAM (Table 3; Fig. 4). This may indicate a preferential substitution of arabinosyl sugars, as was the case with cold-acclimated *Miscanthus* cultivars (Domon *et al.*, 2013). A 10-fold increase in the esterification peak of 42 d VTZ as opposed to 42 d SAM also indicates enhanced cross-linkage with ferulic and *p*-coumaric hydroxycinnamic acids, and a stiffening of the matrix (Hatfield *et al.*, 2016) linked to enhanced freezing tolerance (Rajashekar and Lafta, 1996).

Interestingly, there was an increase in pectin esterase (BAJ96804.1), polygalacturonase (EMT00190.1), and a GDSL esterase (EMT02121.1) in the SAM and VTZ cold-acclimated apoplast (Table 3). The low, SAM methyl-esterified peak area in comparison with control conditions (Table 5) could be due to the increase in pectin esterase inhibitor (BAJ98765.1) abundance (Table 4). Traditional analysis of type II cell walls indicates that pectins comprise a relatively small fraction (<5%) of the cell wall in comparison with glucuronoarabinoxylans; however, these measurements are traditionally based on leaf or stem tissue (Carpita, 1983; Hatfield *et al.*, 2016). Recent experiments with spring wheat ‘Recital’ identified high tissue-specific homogalacturonan deposition in the seed coat (Chateigner-Boutin *et al.*, 2014), and rhamnogalacturonan I was identified to be important in maintaining cell wall integrity during rapid cell expansion in spring wheat ‘Cadensa’ endosperm during germination (Palmer *et al.*, 2015). Baldwin *et al.* (2014) concluded that frost tolerance in pea (*Pisium sativum* L.) was associated with increased pectin and a higher methyl esterification ratio. Since there has not yet been a definitive cell wall compositional study of winter wheat crown tissues, it is possible that methyl-esterified pectins, in conjunction with other cell wall matrix components such as increased concentrations of neutral sugars, enhance cell wall strength.

The observation that: (i) apoplastic fluids containing peroxidases and germin-like proteins with oxalate oxidase activity preferentially accumulated in the 42 d VTZ; (ii) enhanced VTZ methyl esterification in comparison with the 42 d SAM; and (iii) evidence of low temperature-induced diferulic acid (Wakabayashi *et al.*, 2011) collectively suggest that cold acclimation may result in preferential formation of diferulic wall linkages in the VTZ. If so, this would increase cell wall mechanical resistance to ice propagation (Rajashekar and Lafta, 1996). During wall re-organization, glucuronoarabinoxylans exposed by sugar hydrolysis are cross-linked via an ester linkage to ferulic or *p*-coumaric acid (Hatfield *et al.*, 2016). Germin-like proteins with an oxalate oxidase activity (AAG00426.1) as well as peroxidases (EMS52349.1 and EMT19353.1) capable of oxidizing hydrogen peroxide during this cross-linking reaction were shown to increase in abundance in both the SAM and VTZ (Supplementary Table S1 at Dryad; Table 4). Parallels between cross-linkages and subsequent increases in wall rigidity and enhanced freezing tolerance can be drawn with cold response wall rigidity in studies conducted on plants with type I cell walls (Rajashekar and Lafta, 1996; Solecka *et al.*, 2008; Tanino *et al.*, 2013).

Cell wall-related expansins, xyloglucan transglucosylases, and endo-glucanases play a central role in modulating wall extensibility (Cosgrove, 2000; Hatfield *et al.*, 2016). Unlike the decrease in protein abundance of xyloglucan transglucosylases and endo-glucanases (Supplementary Table S1 at Dryad), β-expansins (AJA71651.1 and AAS48872.1) have been identified to increase preferentially in the VTZ during cold acclimation (Table 3). This may seem counterintuitive since expansins weaken non-covalent bonds between wall polysaccharides, facilitating wall extensibility (Cosgrove, 2000). However, β-expansin genes have been identified to be positively cold regulated in Arabidopsis (Lee *et al.*, 2005), improving chilling stress resistance in post-harvest zucchini (*Cucurbita pepo* L. morphotype *Zucchini*) flowers (Carvajal *et al.*, 2015) and postulated as a counterbalance against cold-induced growth-depressing effects in Petunia (*Petunia hybrida*) (Bauerfeind *et al.*, 2015). Conceivably, expansins in the VTZ may play a role in increasing the flexibility of the cell wall during ice accumulation or post-freezing to reduce injury during water influx into the intracellular space.

### Conclusions

After exposure to 4 °C for 42 d, acclimation responses within the SAM and VTZ apoplast diverged. Within the SAM, dehydrin-like proteins act as an anti-desiccant and minimize damage to the membrane during extracellular freeze dehydration (Danyluk *et al.*, 1998). Similarly, SAM proteins with putative vernalization regulatory roles (WCSP2 and WCOR18) were also present to assist in the apical meristem transition from vegetative to floral development through an unknown mechanism (Sasaki *et al.*, 2013). The SAM and VTZ accumulated tissue-specific peroxidases in response to cold acclimation. In the VTZ, the increase in methyl esterification could be associated with increased abundance of peroxidase and Germin B proteins that play a role in peroxide-mediated diferulic cross-linking of glucuronoarabinoxylans (Wakabayashi *et al.*, 2011; Hatfield *et al.*, 2016). These peroxidases act as a defensive response to pathogenic attack in the VTZ post-thawing (Olien and Marchetti, 1976). As suggested by Levitt (1980), when cells such as those found in the SAM are exposed to freezing-induced desiccation, tissue-specific peroxidases are expected to play a greater role in the oxidation of reactive oxygen species to minimize damage to the plasma membrane or other apoplast proteins.

While specific AFPs and pathogenesis-related proteins accumulated in both SAM and VTZ tissues, they were found in greater abundance in the VTZ at 42 d of cold acclimation, which would result in significantly reduced rates of ice migration. Cell wall invertases, fructan exohydrolases, and certain lipid transfer proteins associated with increased accumulation of apoplast sugars preferentially increased in the VTZ. Accumulation of VTZ proteins that directly or indirectly reduce ice propagation and re-crystallization rates are required since the region has the greatest amount of free water (Gusta, 1975) and is the initial site of frost injury (Tanino and McKersie, 1985) within the crown. This differential freezing response, in which the VTZ mitigates and controls the region of damage while the SAM avoids freezing and desiccation injury relative to the VTZ, adds new support to the theory of extra-organ freezing within ‘Norstar’ winter wheat crowns.

Our study has demonstrated that the combination of FTIR-FPA and shotgun proteomics is a useful method for analyzing and localizing cold acclimation-induced tissue-specific accumulation of cell wall-modifying and defense proteins. We have demonstrated that the combined use of proteomics and FTIR-FPA is a useful, comprehensive system to localize cold hardiness traits spatially in individual plants or a series of cultivars. Future advancements in the understanding of tissue-specific cold acclimation mechanisms in winter cereals will be particularly useful to plant breeders intent on improving winter hardiness in these crops.

## Data deposition

The following tables and figures are available at the Dryad Digital Repository: http://dx.doi.org/10.5061/dryad.p65dp

Table S1. List of identified and quantified proteins.

Table S2. Malate dehydrogenase specific activity in ‘Norstar’ SAM and VTZ extracts.

Fig. S1. Venn diagrams of 42/0 d and 21/0 d cold-responsive proteins in the SAM and VTZ.

Fig. S2. Second derivative of absorbance spectra collected from the shoot apical meristem (SAM) and vascular transition zone (VTZ).

## Abbreviations:

AFP: anti-freeze protein
CWMP: cell wall-modifying protein
FTIR: Fourier transform infrared spectroscopy
FPA: focal plane array
LT_50_: lethal temperature at which 50% of the plants recover
SAM: shoot apical meristem
TTC: tetrazolium chloride
VTZ: vascular transition zone
